# Genomic Characterization of VIM and MCR Co-Producers: The First Two Clinical Cases, in Italy

**DOI:** 10.3390/diagnostics11010079

**Published:** 2021-01-06

**Authors:** Vittoria Mattioni Marchetti, Ibrahim Bitar, Mario Sarti, Elena Fogato, Erika Scaltriti, Chiara Bracchi, Jaroslav Hrabak, Stefano Pongolini, Roberta Migliavacca

**Affiliations:** 1 Department of Microbiology, Faculty of Medicine and University Hospital in Pilsen, Charles University, 306 05 Pilsen, Czech Republic; vittoria.mattionimarche01@universitadipavia.it (V.M.M.); jaroslav.hrabak@lfp.cuni.cz (J.H.); 2 Biomedical Center, Faculty of Medicine, Charles University, 323 00 Pilsen, Czech Republic; 3 Clinical Microbiology, Azienda Ospedaliero-Universitaria di Modena, 411 25 Modena, Italy; m.sarti@ausl.mo.it; 4 Laboratory of Clinical Microbiology, ASP “Golgi-Redaelli”, 201 46 Milan, Italy; e.fogato@golgiredaelli.it; 5 Risk Analysis and Genomic Epidemiology Unit, Istituto Zooprofilattico Sperimentale della Lombardia e dell’Emilia-Romagna, 43126 Parma, Italy; erika.scaltriti@izsler.it (E.S.); chiara.bracchi@izsler.it (C.B.); stefano.pongolini@izsler.it (S.P.); 6 Department of Clinical-Surgical, Diagnostic and Pediatric Sciences, Unit of Microbiology and Clinical Microbiology, University of Pavia, 27100 Pavia, Italy; roberta.migliavacca@unipv.it

**Keywords:** colistin resistance, *mcr-4.3*, *mcr-9*, *bla*_VIM-1_, *Enterobacter cloacae* complex

## Abstract

Background: the co-production of carbapenemases and *mcr*-genes represents a worrisome event in the treatment of *Enterobacteriaceae* infections. The aim of the study was to characterize the genomic features of two clinical *Enterobacter cloacae* complex (ECC) isolates, co-producing VIM and MCR enzymes, in Italy. Methods: species identification and antibiotic susceptibility profiling were performed using MALDI-TOF and broth microdilution methods, respectively. Transferability of the *bla*_VIM-_ and *mcr*- type genes was verified through conjugation experiment. Extracted DNA was sequenced using long reads sequencing technology on the Sequel I platform (PacBio). Results: the first isolate showed clinical resistance against ertapenem yet was colistin susceptible (EUCAST 2020 breakpoints). The *mcr-9.2* gene was harbored on a conjugative IncHI2 plasmid, while the *bla*_VIM-1_ determinant was harbored on a conjugative IncN plasmid. The second isolate, resistant to both carbapenems and colistin, harbored: *mcr-9* gene and its two component regulatory genes for increased expression on the chromosome, *mcr-4.3* on non-conjugative (yet co-transferable) ColE plasmid, and *bla*_VIM-1_ on a non-conjugative IncA plasmid. Conclusions: to our knowledge, this is the first report of co-production of VIM and MCR in ECC isolates in Italy.

## 1. Introduction

The ECC (*Enterobacter cloacae* complex) is composed of six species including *E. cloacae* and subspp, *E. kobei*, *E. nimipressuralis*, *E. ludwigii*, *E. asburiae* and *E. hormaechei* [1]. Carbapenem resistant *E. cloacae* complex (CREC) prevalence has increased significantly during recent years [2]. While colistin is considered as the last resort antibiotic for treating infections due to multi-drug resistant strains, increased reports of plasmid mediated *mcr* genes coding for colistin resistance in *Enterobacterales* represent a challenging and alarming situation [3]. Until now, ten variants of the *mcr* gene, *mcr-1*-*mcr-10*, have been identified [4]. The *mcr-4.3* was reported for the first time in Singapore in 2014 on a ColE10 plasmid from a clinical *E. cloacae* isolate [5], and MCR-9 was initially described in 2010 in USA, in a clinical *Salmonella enterica* isolate [6]. On the other hand, among carbapenemase producers, the first detection of metallo-β-lactamase VIM-1 enzyme, was reported in a *Pseudomonas aeruginosa* strain isolated in 1997, in Italy [7]. Up until 30 September, 2020, 73 *bla*_VIM_ variants were overall reported in the National Database of Antibiotic Resistant Organisms (https://www.ncbi.nlm.nih.gov/pathogens/antimicrobial-resistance/).

In Italy, several reports of VIM-producing *Enterobacterales* strains [8,9,10] have been described, with surveillance studies highlighting a minor spread, preceded by KPC, NDM and OXA-48 producers [11,12,13]. Moreover, reports show the prevalence of MCR-1 producers in clinical Italian settings [14,15] and sporadic reports of MCR-4 producing *Salmonella enterica* and *E. coli* strains [16,17]. Nevertheless, no reports of VIM and MCR co-production have yet been reported.

Here we report the first two Italian clinical cases, involving ECC isolates co-producing: VIM-1 and MCR-9 in the first case and VIM-1, MCR-9 and MCR-4.3 in the second one.

## 2. Materials and Methods

### 2.1. Case Presentation, Antimicrobial Susceptibility Test and Molecular Investigations

The first strain, *Enterobacter cloacae* (ENCL_3849), was isolated from a 91 years old female patient admitted to “Istituto Geriatico Milanese” on the 2nd of March 2017 in Milan, Italy. The patient suffered from chronic health complications such as Type II diabetes mellitus, hypothyroidism, sever bilateral gonarthrosis and a risk of falls. From March until the end of June two courses of ceftriaxone were given. On the 7th of July, the blood culture was positive for a multidrug resistant (MDR) *Enterobacter cloacae*. The blood culture was repeated on the 13th and was still positive. On the 16th, the patient suffered from hyperpyrexia and hypotension and was treated with piperacillin. The patient was discharged on the 8th of August. The second strain, *Enterobacter kobei* (ENCB_IB2020), was isolated from a rectal swab (for routine screening purposes) of a 56 years old male patient on the 14th of December 2019 in Modena, Italy.

The species identification was confirmed through matrix-assisted laser desorption ionization-time of flight mass spectrometry (MALDI-TOF MS) using MALDI Biotyper software (Brucker Daltonics, Bremen, Germany). Carbapenemase production was confirmed by meropenem hydrolysis assay [13], while antimicrobial susceptibility profiles were obtained by Microscan AutoScan-4 (Beckman-Coulter) and interpreted in accordance with EUCAST 2020 clinical breakpoints v.10.0 (https://www.eucast.org/fileadmin/src/media/PDFs/EUCAST_files/Breakpoint_tables/v_10.0_Breakpoint_Tables.pdf). Colistin MICs were confirmed through broth-microdilution. Production of class B, D, and A carbapenemases was evaluated using disk combination synergy tests with meropenem and EDTA, temocillin and phenylboronic acid, as inhibitors [17,18,19], respectively. The presence of carbapenemase genes and *mcr* genes were confirmed by polymerase chain reaction (PCR) as described elsewhere [4,20].

### 2.2. Conjugation/Transformation Assay

The ability of the plasmids harboring the *mcr* genes and the *bla*_VIM-1_ gene to conjugate was tested through conjugation experiments. The conjugation was performed in Mueller Hilton (MH) broth (OXOID, Hampshire, UK) using *E. coli* A15^r^Azi as the recipient.

Transconjugants for ENCL_3849 were selected on MH agar (OXOID, Hampshire, UK) plates supplemented with sodium azide (150 mg/L) (Sigma-Aldrich, St. Louis, MO, USA) and ampicillin (1000 mg/L) (Sigma-Aldrich, St. Louis, MO, USA). For ENCB_IB2020, transconjugants were selected on MH agar plates supplemented with sodium azide (150 mg/L), meropenem (2 mg/L) (Sigma-Aldrich, St. Louis, MO, USA) and colistin (2 mg/L) (Sigma-Aldrich, St. Louis, MO, USA). The presence of *bla*_VIM-1_, and *mcr*-like genes in the transconjugants was confirmed through PCR. MICs for transconjugants were performed using the broth-microdilution method. Isolates that failed to transfer the *mcr* genes of interest through conjugation were subjected to transformation; plasmids were extracted using Qiagen Maxi kit (Qiagen, Hilden, Germany) and the competent *E. coli* DH5α cells were used as the recipient. Transformants were selected on MH agar (OXOID, Hampshire, UK) with 2 mg/L colistin. Transformants were confirmed to be MCR producers through PCR.

### 2.3. Whole-Genome Sequencing (WGS)

For genomic characterization, genomic DNA was extracted using NucleoSpin Microbial DNA kit (Macherey-Nagel, Duren, Germany) and sheared using the Hydropore-long on Megaruptor 2 (Diagenode). Microbial multiplexing library preparation was performed without size selection according to the manufacturer’s instructions. The multiplexed library was sequenced using long reads sequencing technology using the Sequel I platform (Pacific Biosciences, Menlo Park, CA, USA) for a 10 h movie run.

### 2.4. Whole-Genome-Sequencing-Data Analysis

Assembly was performed using the “Microbial Assembly” pipeline offered by the SMRT Link v9.0. with the default settings (minimum seed coverage of 30X). In-silico multilocus sequence typing of the strains (MLST) and of the plasmids when applicable (pMLST) was performed; antibiotic resistant genes, plasmid replicons and integrons were detected upon uploading the assemblies to PubMLST (https://pubmlst.org/organisms/enterobacter-cloacae), Plasmid MLST [21], ResFinder 4.1 and CARD [22,23], PlasmidFinder 2.1 [24] and INTEGRALL [25] respectively. BRIG v.0.95 was used to produce figures of comparison of the circular plasmids’ sequences. Genome annotation was done using the NCBI Prokaryotic Genome Annotation Pipeline (PGAP). Species identification of the isolates were confirmed with the NCBI database upon submitting the sequences to GenBank.

## 3. Results

### 3.1. Isolates Susceptibility Profiles

Both strains showed resistance against ampicillin, cefotaxime, ceftazidime, and piperacillin-tazobactam. Moreover, ENCB_IB2020 showed clinical resistance to carbapenems and colistin while ENCL_3849 was susceptible to colistin and resistant to ertapenem. The minimum inhibitory concentrations (MICs) of two strains are shown in Table 1.

### 3.2. Plasmid Transferability

For ENCL_3849, the conjugation experiment was successful and MALDI-TOF species identification and PCR of *bla*_VIM-1_ and *mcr-9* on the transconjugants confirmed the results. Moreover, the transconjugant showed similar antibiotic susceptibility profile as the donor. The PBRT kit for plasmid typing confirmed the presence of two incompatibility groups in the transconjugant: IncN and IncHI2. However, the conjugation experiment in ENCB_IB2020 was not successful. Transformation of the *mcr* bearing plasmid was successful at a low frequency, however it was not stable and the plasmid was lost upon re-streaking.

### 3.3. Whole-Genome Characterization

#### 3.3.1. ENCL_3849

The *E. cloacae* strain ENCL_3849 belonged to sequence type ST382. WGS sequence analysis showed the presence of 6 complete circular contigs; the chromosome (4599410 bp) harbored genes coding for resistance to fosfomycin (*fosA*) and β-lactams (*bla*_ACT-5_). Three un-typable plasmids p3849I (4667 bp), p3849II (4995 bp) and p3846III (190697 bp) did not harbor any antibiotic resistance genes.

**Figure 1:**
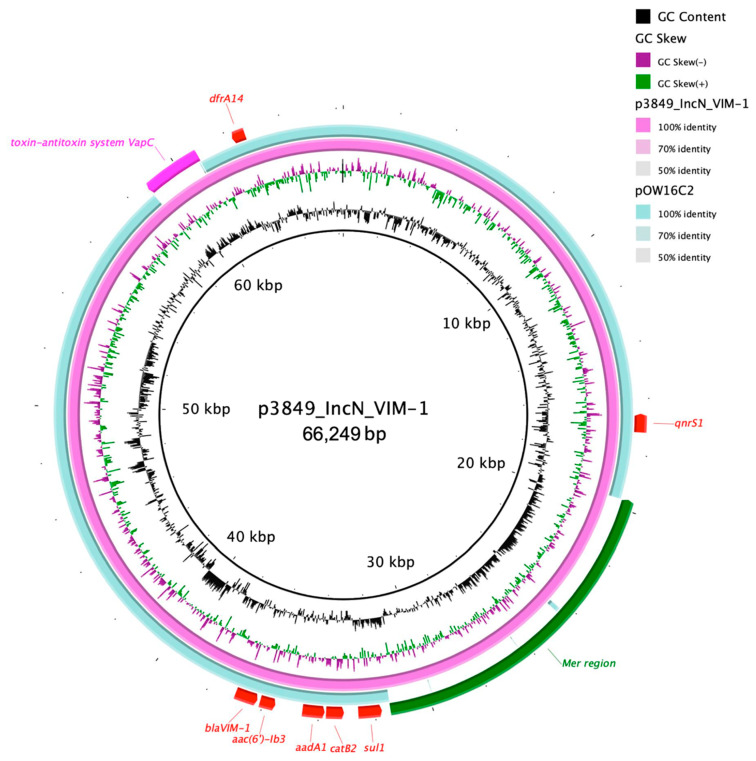
The circular map of p3849_IncN_VIM-1 (pink) against pOW16C2 (turquoise). The outer most curved segments (green, red and purple) correspond to antibiotic resistance genes, the *mer* region, and the toxin-antitoxin system.

Moreover, an IncN plasmid (p3846_IncN_VIM-1; 66,249 bp; pMLST ST7) that harbored genes for resistance against fluoroquinolones (*qnrS1*, *aac(6*′*)-lb3*), aminoglycosides (*aadA1*), trimethoprim (*dfrA14*), sulphonamides (*sul1*), phenicol (*catB2*) and β-lactams (*bla*_VIM-1_) (Table 2). When blasted against the NCBI database, the highest similarity scores corresponded to an IncN (pOW16C2) plasmid (79% sequence similarity, 100% sequence coverage; 59,228 bp; acc. KF977034.1) isolated from a *Klebsiella pneumoniae* strain described in Switzerland. The *bla*_VIM-1_ was found on a novel class 1 integron designated by *In*1128 composing an array of gene cassettes including *bla*_VIM-1_, *aacA4′*, *aphA15*, *aadA1cb*, and *catB2*. Nevertheless, p3846_IncN_VIM-1 had two more DNA cassettes coding for: Type II toxin/antitoxin system (bound with IS26 upstream and a transposase on the other end), and a set of *mer* genes (*merR-T-P-C-D-E*) bound with an IS3 transposase (Figure 1).

**Figure 2:**
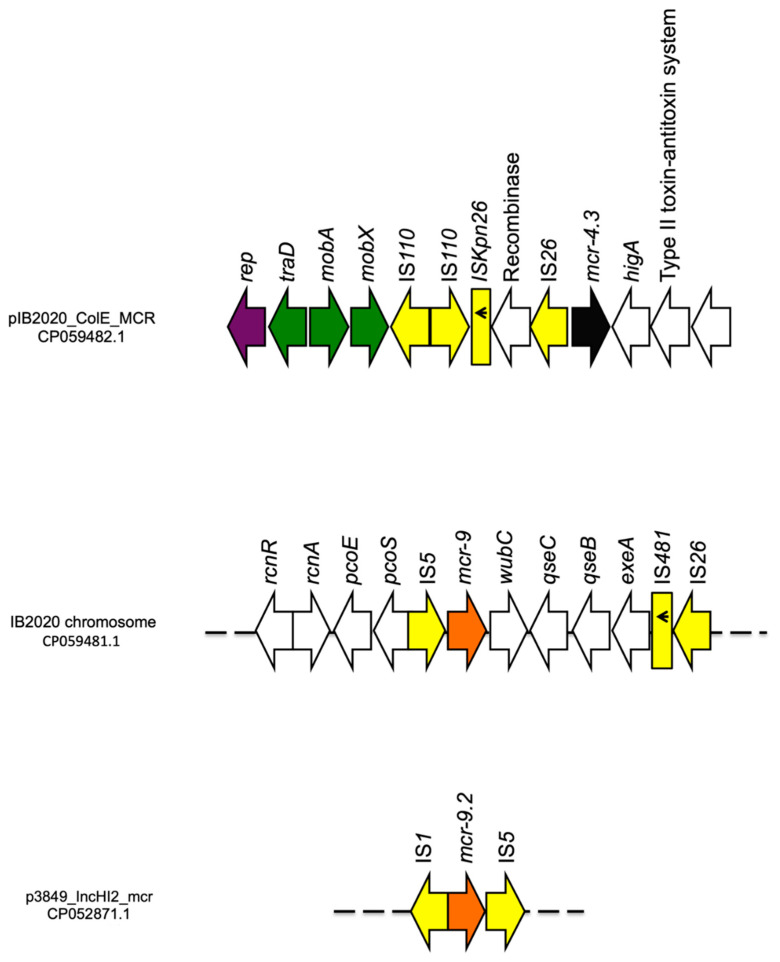
Linear map of the pIB2020_ColE_MCR, *mcr-9* genetic environment of the IB2020 chromosome, and the genetic environment of *mcr-9.2* harbored on p3849_IncHI2_mcr. Arrows show the direction of transcription of ORFs while rectangles represent truncated ORFs. Replication, mobile elements, *mcr-4.3*, *mcr-9* and *mcr-9.2*, transfer/mobility and other remaining genes are designated by violet, yellow, black, orange, and white, respectively.

**Figure 3:**
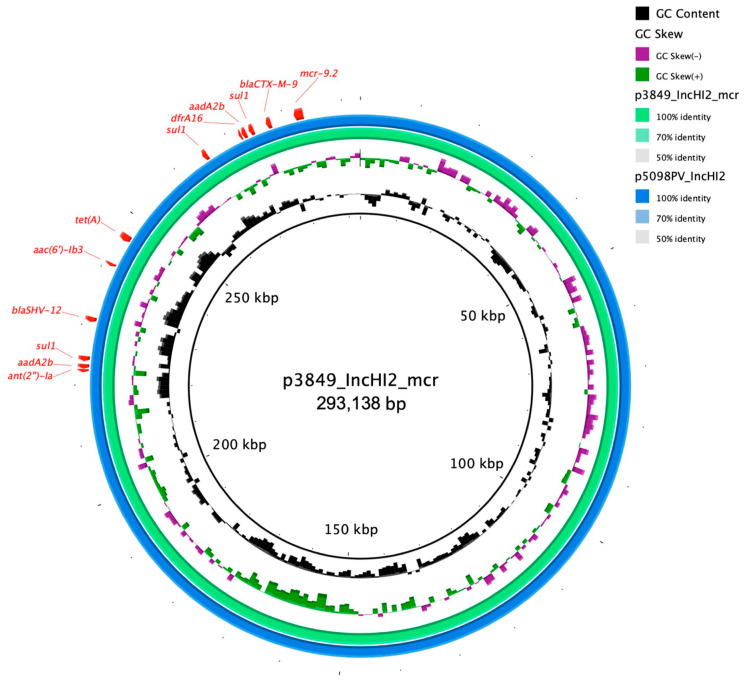
The circular map of p3849_IncHI2_mcr (green) against p5098PV_IncHI2 (blue). The outer most curved segments; red, correspond to antibiotic resistance genes.

Finally, an IncHI2/IncHI2A plasmid (p3846_IncHI2_mcr; 293,138 bp; pMLST ST1) that harbored genes coding for resistance against fluoroquinolones (*qnrA1*, *aac(6′)-lb3*), colistin (*mcr-9.2*), aminoglycoside (*aadA2b* (×2), *ant(2″)-la*), trimethoprim (*dfrA16*), sulphonamide (*sul1*(×3)), tetracycline (*tet(A)*) and β-lactams (*bla*_SHV-12_, *bla*_CTX-M-9_) (Table 2). The *mcr* gene was bound by IS1 and IS5 in opposite orientation (Figure 2). When blasted, p3846_IncHI2_mcr showed high similarity scores with the plasmid p5098PV_IncHI2 (99.99% sequence similarity, 100% sequence coverage; 298,499 bp; acc. CP061512) isolated from *Mixta calida* in Italy (Figure 3).

#### 3.3.2. ENCB_IB2020

The *E. kobei* strain ENCB_IB2020 belonged to ST54. WGS analysis yielded 7 complete circular contigs; the chromosome (4,997,888 bp) that harbored genes coding for resistance against fosfomycin (*fosA*), colistin (*mcr-9*) and β-lactams (*bla*_ACT-9_). The chromosome mediated *mcr-9* was bound by an IS5 upstream in the same orientation, and was followed by the *wbuC* gene (cupin fold metalloprotein), two component-system regulatory genes (*qseC*, *qseB*) responsible for the expression of colistin resistance [26], followed by a truncated IS481 and an IS26 (Figure 2). Three un-typeable plasmids (pIB2020_S; 2020 bp, pIB2020_N; 6127 bp, pIB2020_L; 150,133 bp) and an IncFIB plasmid (pIB2020_IncFIB; 85,067 bp) did not harbor any antibiotic resistant genes.

Moreover, a ColE plasmid (pIB2020_ColE_MCR; 12,808 bp) that harbored *mcr-4.3* gene coding for colistin resistance was found. The *mcr-4.3* gene was bound with an IS26 upstream in opposite orientation followed by a Type II toxin/antitoxin system downstream. Moreover, the plasmid harbored *mobA* and *mobX*, two genes responsible for the plasmid mobility/co-transferability with a conjugative plasmid as reported elsewhere [27] (Figure 2).

**Figure 4:**
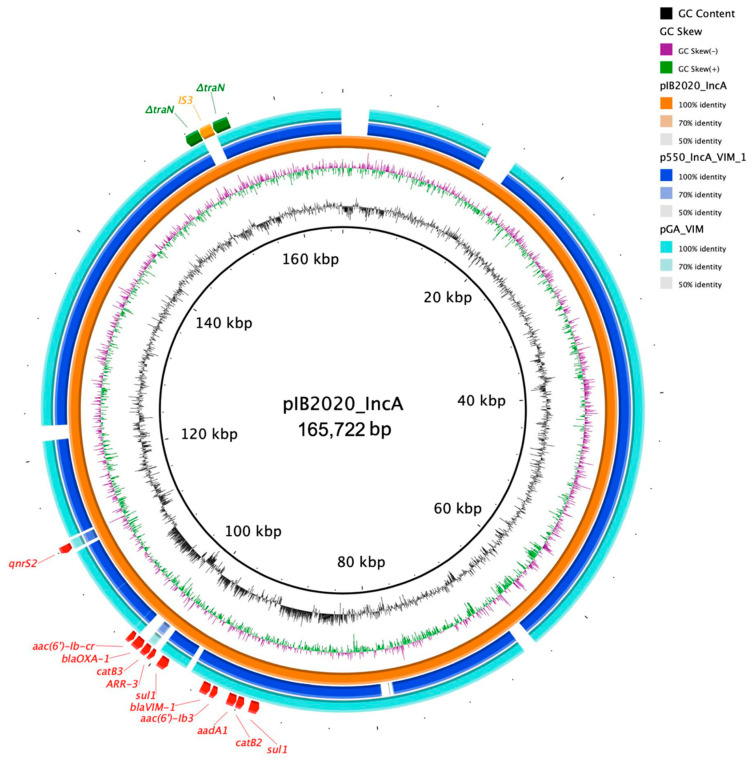
The circular map of pIB2020_IncA (orange) against p550_IncA_VIM_1 (blue) and pGA_VIM (turquoise). The outer most curved segments; red, green and orange, correspond to antibiotic resistance genes, *traN* and IS3, respectively.

An IncA plasmid (pIB2020_IncA; 165,722 bp; pMLST ST12) harbored genes coding for resistance against fluoroquinolones (*qnrS2*, *aac(6′)-lb-cr*, *aac(6′)-lb3*), aminoglycosides (*aadA1*), sulphonamide (*sul1* (2×)), phenicol (*catB3*, *catB2*), rifampicin (*arr-3*) and β-lactams (*bla*_OXA-1_, *bla*_VIM-1_) (Table 2). The *bla*_VIM-1_ determinant was found on the class 1 integron *In*916, composing an array of gene cassettes of *bla*_VIM-1_, *aacA4′*, *aphA15*, *aadA1b*, and *catB2*. *In*916 was involved in the dissemination of the *bla*_VIM-1_ gene in Italy, as previously described [28,29]. When blasted, pIB2020_IncA showed high similarity with pGA_VIM and p550_IncA_VIM_1 (92% and 91% sequence similarity, 100% sequences coverage; 162,608 bp and 1,820,216 bp; CP058224.1 and MN783743.2 respectively) [11,29]; both isolated from *E. coli* strains in Italy (Figure 4). The plasmid pIB2020_IncA was not conjugative; we hypothesize that the interruption of the *traN* gene (coding for conjugation protein) by an IS3 (splitting the gene into two parts) could be the possible reason behind the failure of this plasmid to conjugate.

## 4. Discussion and Conclusion

The reports of VIM and MCR co-production in ECC isolates are increasing, as described elsewhere [30,31,32,33,34]. The presence of *mcr-9*-like genes bound by two insertion sequences as in p3849_IncHI2_mcr and as reported in Sadek et al. 2020 and Bitar et al. 2020 will not express colistin resistance and such plasmids can circulate silently until detected. On the other hand, IB2020 had *mcr-9* on the chromosome in this gene’s cassette: *mcr-9-wubC-qseC-qseB-exeA.* This gene’s cassette was detected to express colistin resistance as presented in our IB2020 isolate [32,34]. Our results confirm and highlight some important aspects: the presence of *mcr-9* determinants together with the two-component regulatory genes, can increase the gene expression, leading to colistin resistance whether on a plasmid or on the chromosome. The association of metallo-β-lactamases and increased colistin resistance largely reduce the numbers of therapeutic options available against severe Gram-negative infections. In particular, with the new combination strategies approved by the US Food & Drug Administration (FDA), the only available options against severe infections are Aztreonam-Avibactam and Cefiderocol, not degraded by metallo-β-lactamases [35,36]. This is evident from the MICs threat that limited the therapeutic option in our case to few antibiotics (Table 1). The presence of *mcr-4.3* encoding ColE plasmid and another *mcr-* gene (*mcr-9*), is alarming. The ColE plasmid also harbored the genes necessary for its mobilization/co-transfer, which indicates that this plasmid is able to co-transfer with a conjugative plasmid, leading to its further dissemination. Moreover, the IncA and IncN plasmids represent self-conjugative plasmids, with a high tendency to acquire different antibiotic resistance islands, which may eventually lead to extremely drug resistant phenotypes and to the spread of different resistance genes in heterogeneous plasmid environment [36,37,38].

Finally, to our knowledge, these are the first cases of *mcr-4.3*, *mcr-9*, *bla*_VIM-1_ and *mcr-9.2* and *bla*_VIM-1_ genes in ECC strains isolated from clinical cases, in Italy.

## Figures and Tables

**Table 1:** Antibiotic susceptibility profiles of the *E. cloacae* 3849 (ENCL_3849) and its transconjugant *E. coli* A15*3849 and ENCB_IB2020 carrying *bla*_VIM-1_ and *mcr* genes.

Isolate	AMP	AMS	ATM	CTX	TET	CAZ	COL	TZP	ETP	MEM	GEN	TOB	TGC	SXT	PIP
*E. cloacae* 3849	>128	>128	>16	>8	>32	>16	0.25	64	>2	0.5	8	>8	0.5	>4	>128
*E. coli* A15*3849	>128	>128	>16	>8	>32	>16	0.25	64	>2	0.5	8	>8	0.25	>4	>128
*E. kobei* IB2020	>128	>128	1	>8	2	>16	8	64	>2	16	0.5	4	0.25	0.5	>128

AMP, ampicillin; AMS, ampicillin/sulbactam; ATM, aztreonam; CTX, cefotaxime; TET, tetracycline; CAZ, ceftazidime; COL, colistin; TZP, piperacillin-tazobactam; ETP, ertapenem; MEM, meropenem; GEN, gentamycin; TOB, tobramycin; TGC, tigecycline; SXT, trimethoprim/sulfamethoxazole; PIP, piperacillin.

**Table 2:** Whole-genome characterization (WGS) analysis of the two MCR/VIM co-producing isolates from Italy.

ID	Species	MLST	Genetic Element	Replicon	pMLST	Antibiotic Resistance Genes
ENCB_IB2020 (2020/8240)	*E. kobei*	ST 54	Chromosome	NA	NA	*fosA, mcr-9*, *bla*_ACT-9_
			pIB2020_IncA	IncA	ST 12	*aac(6′)-lb-cr*, *aac(6′)-lb3*, *qnrS2*, *aadA1*, *sul1* *, *arr-3*, *catB2*, *catB3*, *bla*_OXA-1_, *bla*_VIM-1_
			pIB2020_ColE_MCR	ColE	-	*mcr-4.3*
			pIB2020_IncFIB	IncFIB	-	-
			pIB2020_L	NT	-	-
			pIB2020_N	NT	-	-
			pIB2020_S	NT	-	-
ENCL_3849	*E. cloacae*	ST 382	Chromosome	NA	NA	*fosA*, *bla*_ACT-5_
			p3846_IncHI2_mcr	IncHI2	ST 1	*qnrA1*, *aac(6′)-lb3*, *ant(2′’)-la*, *mcr-9.2*, *aadA2b* *, *sul1* *, *dfrA16, tet(A)*, *bla*_CTX-M-9_, *bla*_SHV-12_
			p3846_IncN_VIM-1	IncN	ST 7	*qnrS1*, *aac(6′)-lb3*, *aadA1*, *sul1*, *dfrA14*, *catB2, bla*_VIM-1_
			p3846III	NT	-	-
			p3849I	NT	-	-
			p3849II	NT	-	-

NT: not typable; NA: not applicable; *: multiple copies; - : none

## Data Availability

The nucleotide sequences of the chromosome and plasmids of ENCB_IB2020 and ENCL_3849 were deposited in GenBank and the following accession numbers have been assigned respectively: CP059480-CP059486 and CP052870-CP052875.
